# The Stability of TSH, and Thyroid Hormones, in Patients Treated With Tablet, or Liquid Levo-Thyroxine

**DOI:** 10.3389/fendo.2021.633587

**Published:** 2021-03-10

**Authors:** Alessandro Antonelli, Giusy Elia, Francesca Ragusa, Sabrina Rosaria Paparo, Gabriella Cavallini, Salvatore Benvenga, Silvia Martina Ferrari, Poupak Fallahi

**Affiliations:** ^1^ Department of Surgical, Medical and Molecular Pathology and Critical Care, University of Pisa, Pisa, Italy; ^2^ Department of Clinical and Experimental Medicine, University of Pisa, Pisa, Italy; ^3^ Interdepartmental Research Centre on Biology and Pathology of Aging, University of Pisa, Pisa, Italy; ^4^ Section of Endocrinology, Department of Clinical and Experimental Medicine, University of Messina, Messina, Italy; ^5^ Master Program on Childhood, Adolescent and Women’s Endocrine Health, University of Messina, Messina, Italy; ^6^ Interdepartmental Program on Molecular & Clinical Endocrinology, and Women’s Endocrine Health, University Hospital, A.O.U. Policlinico Gaetano Martino, Messina, Italy; ^7^ Department of Translational Research of New Technologies in Medicine and Surgery, University of Pisa, Pisa, Italy

**Keywords:** thyroid-stimulating hormone (TSH), hypothyroidism, soft gel capsule L-T4, liquid L-T4, malabsorption, levothyroxine

## Abstract

Approximately, 5% of the population is affected by hypothyroidism, mainly women and persons aged more than 60 years. After the diagnosis of hypothyroidism the usual therapy is tablet levothyroxine (L-T4), with a monitoring of the thyroid-stimulating hormone (TSH) level in primary hypothyroidism every 6–8 weeks and L-T4 is adjusted as necessary to reach an euthyroid state. Once TSH is stabilized in the normal range, it is recommended to conduct annual testing in the treated subjects to warrant suitable replacement. More recently advances regarding L-T4 treatment are the introduction of new oral formulations: the liquid solution, and soft gel capsule. The soft gel capsule permits a quick dissolution in the acid gastric pH. The liquid preparation does not require an acid gastric environment. Many pharmacokinetic studies demonstrated a more rapid absorption for the liquid L-T4, or capsule, than with tablet. Many studies have shown that the liquid, or capsule, formulations can overcome the interaction with foods, drugs or malabsorptive conditions, that are able to impair the tablet L-T4 absorption. Lately studies have suggested that liquid L-T4 can permit to maintain more efficiently normal TSH levels in hypothyroid patients in the long-term follow-up, than tablet L-T4, both in patients with malabsorptive states, and in those without malabsorption. Further large, prospective, longitudinal studies are needed to evaluate the stability of TSH, in hypothyroid patients treated with different L-T4 formulations.

## Introduction

In physiological conditions, thyroid hormones (TH) are produced by the gland and their synthesis depends on normal iodide transport. Approximately 10%–20% triiodothyronine (T3) and 80%–90% thyroxine (T4) are secreted by the thyroid, then T4 is converted to T3 by deiodinase enzymes (1). T4 functions as a pro-hormone, and T3 is about four to five times more potent than T4 (2). TH regulate protein synthesis, energy metabolism, and the sensitivity to other hormones (3).

Thyroid-stimulating hormone (TSH), secreted by the pituitary, regulates TH output from the thyroid, and it is controlled by thyrotropin-releasing hormone (TRH), secreted by the hypothalamus (4). A negative feedback controls the release of TH: TSH secretion is suppressed if free T3 (FT3), or free T4 (FT4) levels are elevated, and the same happens for the production of TRH by the hypothalamus (2).

The synthetic hormone levothyroxine (L-T4) has a structure comparable to T4, and it is done as substitutive therapy of hypothyroidism-associated conditions (2). It is absorbed in duodenum, jejunum and ileum (5).

The frequency of hypothyroidism is higher in women, especially over 60 years, and it can be diagnosed measuring serum TSH and free T4 values (2). Hypothyroidism can be caused by different conditions (6–9): 1) A low iodine intake in countries with severe iodine deficiency; 2) autoimmune thyroiditis that is the most common cause in iodine sufficient countries; 3) thyroidectomy; 4) radioiodine treatment for hyperthyroidism. Drugs, such as tyrosine kinase inhibitors, and immune checkpoint inhibitors, are a novel cause of primary hypothyroidism (10, 11).

The L-T4 daily dose is chosen according to the principal cause of hypothyroidism, the therapeutic target, and the patients’ lean body mass (12). Slight changes in blood levels can lead to treatment failure or iatrogenic thyrotoxicosis (13), for this reason the individualization of oral T4 treatment is necessary.

After the diagnosis of hypothyroidism the usual therapy is tablet L-T4; TSH is monitored in primary hypothyroidism every 6–8 weeks, and L-T4 is adjusted as necessary to reach euthyroidism. The optimal daily L-T4 replacement dosage is 1.5–1.7 μg/kg body weight/day, that can normalize TSH in most hypothyroid patients (14). Anyway, ~20%–50% of patients on L-T4 do not attain a normal TSH in cross sectional studies (15) and need an adjustment of therapy (16), due to various interfering issues (17).

Once the TSH is stabilized in the normal range, it is recommended to conduct an annual testing in treated  subjects to warrant suitable replacement. A study retrospectively evaluated 452 patients treated for hypothyroidism, assessing the number of those who successively had therapeutic and non-therapeutic TSH values 10–14 months later (18). The percentage of normal repeat TSH values significantly decreased with increasing medication dosage (P=0.01). Considering patients whose maintenance dose was <75 μg/day, 90.8% had normal repeated TSH values, in comparison to 77.5% of those needing ≥125 μg/day, who had significantly lower odds of normal repeated TSH [odds ratio, 0.31; 95% confidence interval (CI), 0.13–0.76; P=0.01]. The authors concluded that the dose of TH replacement was predictive of normal TSH values, and patients taking ≥125 μg/day L-T4 are less likely than those needing lower doses to have a normal repeated TSH value in 1 year (18).

More recently, a study investigated whether any clinical predictors exist that can distinguish patients who could be monitored safely, less frequently than yearly (19). Seven hundreds and fifteen patients treated for hypothyroidism, with normal TSH value while taking L-T4, were retrospectively evaluated. L-T4 dosage >125 μg/day had an augmented hazard ratio of 2.4 (95% CI, 1.7–3.4; P<0.0001) for time to first follow-up elevated TSH value, but doses lower than that did not raise the hazard ratio. One year after the first normal TSH, 91.1% of patients receiving ≤125 μg/day had a persisting normal TSH, while only 73.3% of patients taking >125 μg/day did. The authors concluded that patients receiving daily dosages >125 μg/day of L-T4 have more difficulties to maintain stable TSH values over time than those needing lower dosages of L-T4 (19).

The aim of this review is to evaluate the stability of TSH, and TH, in patients treated with tablet, or liquid L-T4.

We searched relevant and recently (from 2000) published papers on PubMed using principally the terms “levothyroxine therapy”, “tablet levothyroxine”, “liquid levothyroxine”, “soft gel capsule L-T4”, “levothyroxine malabsorption”, in combination with “hypothyroidism”, and “TSH”.

## Novel Oral L-T4 Preparations

Some patients are not compliant with the prescribed L-T4 regimen, and this can cause a condition of pseudo-malabsorption. Moreover, resistance to TH (RTH) is a rare autosomal dominant disorder that leads to elevated free TH levels, in the presence of normal or increased serum TSH concentrations, if it is generalized because both the pituitary and peripheral tissues are then partially resistant (20). Once those are excluded, an altered intestinal absorption of L-T4 (caused by gastrointestinal disorders, some nutrients, or drugs) is considered the principal cause of refractory hypothyroidism. Moreover, the case of a 49-year-old patient suffering from hypothyroidism refractory to oral LT-4 substitution after total thyroidectomy and radioiodine therapy for papillary thyroid cancer (PTC) was reported (21). Furthermore, three cases of critically ill patients with prolonged respiratory failure, suppressed mental status and unexplained hypotension were reported, who showed normal or mildly abnormal TSH values and free thyroxine markedly suppressed. After initiation of intravenous L-T4, the patients could be weaned off vasopressors and were successfully extubated shortly thereafter, suggesting that the early recognition and treatment of hypothyroidism in presence of a critical illness can contribute to recovery from hypotension or the need for mechanical ventilation (22). The improperly elevated TSH, caused by such conditions, is managed by the increase of the L-T4 daily dosage, that can cause iatrogenic hyperthyroidism, especially when the underlying disorders (i.e., with a gluten-free diet) are cured, or the effect of interfering drugs is stopped. Patients refractoriness to a “normal dose” of L-T4 (23) has led to novel hormonal formulations that could permit to attain an improved performance of this drug: the liquid formulation and soft gel capsule.

### Liquid L-T4

The liquid formulation is bioequivalent with the traditional one, but it has a shorter mean time to attain the higher concentration than soft gel capsule or tablets (1.96 vs. 2.38 vs. 2.25 h) (24), and it contains L-T4, ethanol, and glycerin; with respect to tablets, it does not require an acid gastric pH to dissolve it (25).

Liquid formulation effectiveness has been evaluated in two meta-analyses. The first meta-analysis suggested that subjects receiving L-T4 tablets with suboptimal TSH values can achieve the desirable TSH following the switch to liquid L-T4 using the same dose (26), and the second indicated that the efficacy of liquid L-T4 is higher (vs. tablets) in patients having/or not malabsorption in replacement or suppressive treatment (27).

The prescription of liquid L-T4 has been evaluated in newborns with congenital hypothyroidism (28). Seventy-eight patients were enrolled, of whom half received liquid L-T4 and the other half L-T4 in tablets. All subjects had similar birth weight, gestational age, etiology, and severity of congenital hypothyroidism, screening TSH, median initial L-T4 dose, and age at onset of therapy. FT4 levels normalized within 10 days of therapy, and TSH within 7–10 days, in 87% of patients receiving the liquid formulation and in 82% administered with tablets. Albeit L-T4 dose and free T4 levels were comparable, patients taking liquid L-T4 had significantly lower TSH values in comparison to those receiving tablets, at 7–10 days (P=0.05) and 6–8 months (P=0.043) of therapy. The authors concluded that the TSH inhibition rate observed with liquid L-T4 could be related to a major absorption with respect to tablets (28). These data were confirmed also by another study (29).

Moreover, it has been shown that the L-T4 solution can control better serum TSH values compared to tablets also in elderly (30), and in pregnant women (31).

### Soft Gel Capsule

Sodium L-T4 is dissolved in water and glycerin in soft gel capsule, and put in a gelatin matrix, to protect the active ingredient from degradation. It does not contain lactose, gluten, alcohol, sugar, or dyes (25).

Soft gel L-T4 was evaluated by few clinical studies. A study evaluated whether soft gel capsule formulation is able to overcome the malabsorption associated with the consumption of coffee (32). Eight patients with this issue were recruited, including one with hypothyroidism. The subjects were switched to capsule at the same L-T4 dosage for 6 months. All patients assumed the capsule with water, on days 1–90 patients followed a proper habit, taking coffee 1h later the drug assumption; whereas on days 91–180 they followed an improper habit by taking coffee ≤5 min later after the capsule ingestion. In seven patients after the switch, TSH values were 0.41 ± 0.46 mU/ml in those following a proper habit vs. 0.28 ± 0.20 pre-switch, and 0.34 ± 0.30 in patients following an improper habit vs. 1.23 ± 1.47 pre-switch (P<0.001). These findings indicated that soft gel capsule is effective in patients who are not able or do not wish to modify their improper habit of taking L-T4 (32).

A study investigated the daily requirement of L-T4 in 103 patients who had undergone to thyroidectomy. The L-T4 dose necessary to reach normal TSH values was similar between the two types of formulations (soft gel capsules and tablets), but a statistically significant decrease of about 28% in the mean TSH was reported with the soft gel in comparison to tablets (33).

Recently, a study evaluated the effect of switching from tablets to soft gel capsule in hypothyroid patients, without an increased need of L-T4 (34). Hypothyroid subjects with no confirmed malabsorption had an improved TSH after 3 months from the switch. Circulating TSH level was in the normal range in 11/18 patients receiving L-T4 tablets, while after the switch in 16/18, and the median TSH was lower than that obtained with the classical formulation (34).

## Stability of TSH, and TH, in Patients Taking Tablets, or Liquid L-T4

L-T4 is prescribed all over the world as substitutive therapy in case of hypothyroidism, and in patients affected by thyroid cancer after thyroidectomy (35). Even if the substitutive therapy with L-T4 has been used for >60 years, cross-sectional studies demonstrated that 40%–48% of patients receiving L-T4 are under- or over-treated (36, 37).

The recently marketed new formulations of L-T4, in comparison to tablets, lead to a significant decrease in TSH variability in hypothyroidism, in young and old people (30, 38).

### Food Interference

A study compared the TH profile in patients treated with tablets or liquid L-T4 with an enteral feeding tube (29). The day after surgery, 20 euthyroid subjects who had undergone to total laryngectomy and thyroidectomy began L-T4, by an enteral feeding tube. Before administration, tablets were fragmented and enteral feeding was blocked for 30 min before and after L-T4 therapy, while the liquid solution was put immediately in the nasoenteric tube. The findings demonstrated that liquid L-T4 can be done through a feeding tube without the necessity of an empty stomach, ameliorating its administration by nurses (29). The reported data permit to demonstrate that one of the major benefit of liquid L-T4 is that it can be administered in patients unable to swallow capsules or tablets.

The food interference with L-T4 absorption has been evaluated also by other studies, for example the assumption of tablet L-T4 with coffee, or with water and then coffee within few minutes, can lead to an improper TSH response (39). After a casual identification of an euthyroid subject who erroneausly assumed liquid L-T4 with coffee at breakfast, 54 patients were identified, taking the same dose of L-T4 30 min before breakfast. No significant differences in TH levels existed in patients consuming L-T4 at breakfast or 30 min before it for 3 and 6 months (39). Another study enrolled 61 patients, among whom 59 completed it, to compare L-T4 at breakfast or 10 min before it, respect to L-T4 30 min before breakfast, with no clinically relevant differences with respect to the timing of administration (40). Moreover, a placebo-controlled, double-blind trial was conducted in 77 hypothyroid patients, who received randomly the liquid L-T4 at breakfast, or at least 30 min before it, with no significant differences in TSH and TH levels in both cases. The reported data indicated that the liquid preparation can be swallowed at breakfast, favouring the therapeutic compliance (41).

Moreover, a study assessed changes in quality of life (QoL) of 418 hypothyroid patients who were not satisfied with their therapy with L-T4 tablets. One hundred-ten patients (26.3%) complained of the timing of their L-T4 therapy (30–60 min before breakfast), and were switched from tablets taken 30–60 min before breakfast to liquid L-T4 at breakfast. An improved QoL, linked to the easier adherence to therapy, was reported by 66.6% of 102 patients who completed the study after the switch (P<0.01) (42).

### Impaired Gastric Acidity

The presence of Helicobacter Pylori (HP) can negatively impact drugs bioavailability, and the level of gastric pH owing to the inflammatory condition associated with it (43, 44), leading to the unpredictability of the absorbed dosage. Patients with an impaired gastric acid need a higher daily dosage of L-T4, of approximately 22%–34%, in presence of atrophic gastritis, HP-related gastritis, or both (45).

Twenty-eight patients with HP infection and 15 without gastric alterations, treated with the same dosage of L-T4, in tablets for 9 months or oral liquid formulation for 6 months, respectively, were investigated (46). HP infection was eradicated after 3 months of L-T4 treatment. After 3 months (before HP eradication), subjects treated with the liquid formulation had a greater TSH reduction (P=0.029) and a greater homogeneity in the TSH values (P=0.025), with respect to tablets. At 9 months (after 6 months of HP eradication) mean TSH was lower in patients taking L-T4 tablet (P=0.006), while in the group of patients without gastric alterations, no differences were observed, at each time point, in the mean TSH, and TSH variations, between the two L-T4 formulations. The authors concluded that L-T4 liquid preparation can lead to a better clinical response (vs. tablets) in hypothyroid subjects with HP infection (46).

L-T4 malabsorption can occur also in presence of autoimmune atrophic gastritis (47). A study enrolled 391 patients with subclinical or clinical hypothyroidism associated with autoimmune thyroiditis administered with L-T4, and screened circulating parietal cell antibodies (PCA) as marker of autoimmune gastritis (48). A higher L-T4 requirement was shown in patients with positive PCA vs. those with negative PCA (1.24 ± 0.40 μg/kg vs. 1.06 ± 0.36 μg/kg). Among patients with positive PCA, a higher dose of daily L-T4 was reported in those with proven gastritis in comparison to those without gastric damage (1.52 ± 0.40 μg/kg vs. 1.15 ± 0.33 μg/kg) (P<0.0001) (48).

Five patients with autoimmune gastritis, and hypothyroidism, were evaluated in a case series. Patients received L-T4 tablets, and upon the switch to the same dosage of liquid L-T4, circulating TSH normalized in all patients. Among them, four patients were switched back to L-T4 tablets at the same dosage, and TSH worsened again. It was concluded that liquid L-T4 can bypass the altered gastric pH associated with atrophic gastritis (49).

The proton pump inhibitors (PPIs) bind covalently to the H^+^/K^+^ATPase enzyme and this can block the secretion of gastric acid (50). A prospective cohort study was conducted in 24 patients, in whom L-T4 therapy failed after L-T4 and PPIs concurrent assumption. Following the switch from L-T4 tablets to the liquid formulation, at the same daily dose, a significant decrease in serum TSH was shown, even maintaining the co-ingestion of PPIs (51).

### Intestinal Malabsorption

Drug malabsorption can derive from bariatric surgery (52, 53). A study evaluated 17 hypothyroid patients [who had received L-T4 tablets for >1 year prior surgery; four biliary pancreatic diversions (BPD); 13 Roux-en-Y gastric bypasses (RYGB)] with elevated TSH levels from 3 to 8 months after surgery. TSH significantly decreased following the switch from tablets to liquid L-T4 (30 min before breakfast, at the same dose), in patients submitted to RYGB, or BPD, in this way circumventing the issue of malabsorption in BPD-treated patients, and confirming preceding findings obtained in patients undergone to RYGB (52). Another study (53) reported four hypothyroid patients who were in euthyroidism with L-T4 tablets before RYGB, and whose TSH levels increased after surgery. Switching from tablets to the liquid formulation, TSH declined. These data permit to hypothesize that liquid L-T4 can bypass the malabsorption linked to bariatric surgery (52, 53).

Among the gastrointestinal diseases that lead to L-T4 malabsorption, lactose intolerance (LI) is of great interest. In case of LI, a low lactose diet should be used, such as a lactose-free L-T4 preparation, in order to re-establish euthyroidism, without increasing the necessary dose of L-T4 (54, 55).

A cohort study analyzed the necessary L-T4 dosage in 34 hypothyroid subjects with LI, but not compliant with a lactose-free diet (56). The target TSH was attained with a median L-T4 of 1.31 μg/kg/day in hypothyroid patients. In subjects with LI, 5/34 reached the desired TSH with 1.29 μg/kg/day L-T4 (a similar dose), whereas 29/34 needed a gradually augmented L-T4 dosage and the target TSH was achieved at a median L-T4 of 1.81 μg/kg/day (P<0.0001). Among them, six patients had also other gastrointestinal disorders, and needed L-T4 at the dose of 2.04 μg/kg/day. In the other 23 patients with isolated LI, a median L-T4 dosage of 1.72 μg/kg/day (P<0.0001) was necessary to achieve target TSH levels. These data showed that in presence of LI the necessity for oral L-T4 in hypothyroid patients increased significantly (56).

Moreover, in 5 patients with hypothyroidism and LI, the switch from L-T4 tablets with lactose to a liquid preparation without lactose at the same dose normalized serum TSH values (57). In the 1^st^ patient, after 1 month from the switch to liquid L-T4, TSH was in the normal range, and it increased again with the re-administration of L-T4 tablets at the same dosage for 4 weeks. In the 2^nd^ patient, the TSH level was elevated with L-T4 tablets 150 μg daily, and upon 1 month from the switch to the liquid formulation it was in the normal range. To investigate the relationship between TSH normalization and the oral preparation, L-T4 tablets was re-administered for 4 weeks at the same dose, and circulating TSH worsened again. In the 3^rd^ patient, the L-T4 dose was increased, with no correction of hypothyroidism. Then the patient was switched to a liquid formulation and TSH was in the normal range after 1 month. Once L-T4 tablets were re-administered for 4 weeks, serum TSH increased again. The 4^th^ patient received L-T4 tablets after thyroidectomy for PTC and radioiodine treatment. Owing to LI, the dose of L-T4 was augmented but hypothyroidism was not corrected. The patient was switched to liquid L-T4 at the same dose, and TSH values normalized. Following 2 months, TSH was suppressed, and it was maintained suppressed through time. The 5^th^ patient, with autoimmune thyroiditis and hypothyroidism, was switched to liquid L-T4, owing to LI, and upon 2 months, TSH levels were in the normal range. The reported data demonstrated that the liquid L-T4 preparation permitted a better control of TSH, and when TSH, FT3, FT4 were measured again after 6 months, still resulted in the normal range in all the 5 cases (57).

Celiac disease (CD) is another important cause of L-T4 malabsorption. It is an immune-mediated enteropathy, caused by the ingestion of wheat gluten in genetically predisposed subjects (58). A gluten-free diet can improve TSH levels in patients with CD, indicating the importance of the impairment of the intestinal barrier in this disease. A study evaluated the necessary L-T4 dose in 35 patients with hypothyroidism, chronic autoimmune thyroiditis and atypical CD, reporting the need of an increased dose of L-T4, reversed by a gluten-free diet or by increasing T4 dose (59).

Five hundred hypothyroid patients were enrolled in a study and 144/500 needed a L-T4 dose ≥125 μg/day. Nine patients had CD, and 8/9 needed ≥125 μg/day of L-T4. Patients requiring ≥125 μg/day of L-T4 had a significantly higher risk of CD (P<0.001), and CD was found in 5.6% of them (60).

### Patients With No Malabsorption or Drug Interference

The effectiveness of liquid L-T4 was evaluated in hypothyroid patients with no malabsorption or drug interference.

A prospective study enrolled 152 hypothyroid subjects with no malabsorption or drug interference, switched from tablets to liquid L-T4, 30 min before breakfast, at the same dose (61). TSH values significantly declined at the 1^st^ (P<0.05) and the 2^nd^ control (P<0.01), whereas FT4 and FT3 did not change, suggesting a higher effectiveness of liquid L-T4 (than tablets) to control TSH in hypothyroid subjects with no malabsorption, drug interference, or gastric disorders (61).

Moreover, a study enrolled after thyroidectomy 105 patients without malabsorption, of whom 52 received liquid L-T4, and 53 L-T4 tablets, with the same dose (1.5 μg/kg/day) (62). The day after surgery, patients began to take the medication, 30 min before breakfast. Significantly lower TSH values were obtained in patients receiving liquid L-T4, in comparison to those administered with the tablets, at the 1^st^ (P<0.05), and the 2^nd^ control (P<0.01), whereas FT4 and FT3 did not change. Patients treated with L-T4 tablets were more prevalently in the hypothyroid range of TSH (>3.6 μU/ml) (62).

Another study was conducted in 21 hypothyroid subjects with no malabsorption or drug interference, with elevated TSH levels under therapy with L-T4 tablets. Patients were switched to the liquid formulation at the same dose, 30 min before breakfast, and TSH significantly decreased 2 months following the switch (63). Among the 21 patients, 15 switched back to tablets, and TSH raised again to hypothyroid values. All subjects were then administered with the liquid L-T4, and TSH, FT3, and FT4 were measured again following 6 and 12 months, and resulted in the normal range (63). It was concluded that in hypothyroid patients with no malabsorption, drug interference, or gastric disorders, the liquid formulation is better in the control of TSH levels with respect to tablets (62, 63).

The prevalence of aberrant thyroid function has been controversial for a long time. A study evaluated the prevalence of elevated TSH in participants in a statewide fair in Colorado. The prevalence of high values of TSH was 9.5% and of decreased TSH levels of 2.2%. The findings reported that 40% of patients taking thyroid medications had abnormal TSH values (37).

Furthermore, another study evaluated patients treated for differentiated thyroid cancer (DTC), randomly administered with L-T4 in tablets or in liquid solution (36). One hundred and two patients were enrolled, 51 treated with L-T4 tablets and 51 with the liquid formulation, at the dose of 1.9 μg/kg/day, from the day after post-surgery ^131^I treatment. The 1^st^ control was done 8–12 months after ^131^I remnant ablation, and furtherly after 12 months. TSH increased significantly in patients taking tablets in comparison to those receiving the liquid preparation. The authors concluded that liquid L-T4 can lead to a significantly more elevated number of DTC patients with TSH levels in range for the American Thyroid Association (ATA) risk score, decreasing TSH variability through time (36).

More recently, a study compared the stability of TSH in hypothyroid patients taking liquid L-T4 compared to those receiving tablets (64). Five hundred and fifty hypothyroid patients received the liquid formulation, and 225 L-T4 tablets. After 1 year, normal TSH levels were present in 91% of the patients who received the L-T4 liquid solution whereas only in 85% of those taking L-T4 tablets. After 2 years, TSH normal values were attained in 87% of patients who received the L-T4 liquid preparation while only in 76% of those administered with tablets (P<0.05) (64).

## Conclusion

The frequency of hypothyroidism is higher in women, especially over 60 years. It is present in about 5% of the population and it can be diagnosed measuring serum TSH and free T4 values. After the diagnosis of hypothyroidism the usual therapy is tablet L-T4, monitoring TSH values in primary hypothyroidism every 6–8 weeks. Once the TSH is stabilized in the normal range, it is recommended to conduct annual testing on all treated  subjects to warrant suitable replacement.

L-T4 absorption can be impaired by various conditions, such as with some food, or assuming it during breakfast, or conditions of reduced gastric acidity, bariatric surgery, LI, CD, and drugs that alter the gastric pH, avoiding the dissolution of tablets.

New oral L-T4 formulations have been developed: the liquid preparation, and soft gel capsule. The liquid solution does not require an acid gastric environment, and soft gel capsule permits a quick dissolution in the acid gastric pH, demonstrating a more rapid absorption for liquid L-T4, or capsule, than tablets.

Liquid L-T4 Should Be Used Right From the Start of the Treatment in the Following Categories of Hypothyroid Patients:

- with food and beverages (coffee, etc.) interference with tablet L-T4 absorption and not compliant with the abitual ingestion of L-T4 30–60 min before breakfast;
- with malabsorption associated with an increased gastric pH;
- with malabsorption after bariatric surgery, or with intestinal malabsorption;
- with malabsorption induced by interferent drugs;
- with typical or atypical CD, or in patients with LI;
- who are not able to swallow the tablets.

Furthermore, liquid L-T4 should be used in patients with a sub-optimal response to tablets L-T4, and a TSH threshold of >4 μU/ml should be used for switching to the liquid formulation.

Moreover, many studies have suggested that liquid L-T4 permits to maintain more efficiently normal TSH in hypothyroid patients in the long term follow-up, than L-T4 tablets, both in patients with malabsorptive states, than in those without malabsorption.

Further large, prospective, longitudinal studies are necessary to evaluate the stability of TSH, in hypothyroid patients administered with different L-T4 formulations.

## Author Contributions 

AA, SMF, and PF conceived the paper. All authors contributed to the article and approved the submitted version.

## Conflict of Interest

The authors declare that the research was conducted in the absence of any commercial or financial relationships that could be construed as a potential conflict of interest.
